# Evaluation of telemental health services for people with intellectual and developmental disabilities: protocol for a randomized non-inferiority trial

**DOI:** 10.1186/s12913-023-09663-6

**Published:** 2023-07-25

**Authors:** Luther G. Kalb, Jessica M. Kramer, Tawara D. Goode, Sandra J. Black, Susan Klick, Andrea Caoili, Samantha Klipsch, Ann Klein, Micah P. Urquilla, Joan B. Beasley

**Affiliations:** 1 Center for Autism and Related Disorders, Kennedy Krieger Institute, Baltimore, USA; 2 Department of Mental Health, Johns Hopkins Bloomberg School of Public Health, Hampton House, 624 N. Broadway, 8th Floor, Baltimore, MD 21205 USA; 3 University of Florida at Gainesville, Gainesville, USA; 4 Georgetown National Center for Cultural Competence, Washington, USA; 5 Department of Pediatrics, Georgetown University Medical Center, Washington, USA; 6 Department of Social Work, University of New Hampshire, Durham, USA; 7 Institute on Disability/UCEDD, University of New Hampshire, Durham, USA

**Keywords:** Disability, Crisis, Mental health, Telehealth, Autism, Virtual

## Abstract

**Background:**

Roughly 40% of those with intellectual/developmental disabilities (IDD) have mental health needs, twice the national average. Unfortunately, outpatient mental health services are often inaccessible, increasing reliance on hospital-based services. While telemental health services hold potential to address this gap, little is known about the effectiveness of telemental health for the diversity of persons with IDD, especially as it relates to crisis prevention and intervention services. Accordingly, the aims of this study are to: (1) compare telemental health versus in-person crisis prevention and intervention services among people with IDD; and (2) understand if outcomes vary across subpopulations, in order to identify potential disparities.

**Methods:**

This study will take place within START (Systemic, Therapeutic, Assessment, Resources, and Treatment), a national evidence-based model of mental health crisis prevention and intervention for people with IDD. A total of 500 youth and adults, located across nine states, will be randomized 1:1 to telemental health vs. in-person. Participant inclusion criteria are ages 12–45 years, living in a family setting, and newly enrolled (within 90 days) to START. Outcomes will be assessed, using a non-inferiority design, for up to 1 year or until discharge. The intervention is comprised of four components: (1) outreach; (2) consultation/coping skills; (3) intake/assessment; and, (4) 24-hour crisis response. The in-person condition will deliver all components in-person. The telemental health condition will deliver components 1 & 2, via telephonic or other communication technology, and components 3 & 4 in-person. Outcomes include mental health crisis contacts, mental health symptoms, emergency psychiatric service use, perceived quality of mental healthcare, and time to discharge.

**Discussion:**

To our knowledge, this will be the first trial of a telemental health crisis program for the IDD population. The study will be executed by an interdisciplinary team of experts that includes persons with lived experience of disability. Understanding the benefits of specific telemental health methods has important implications to the design of interventions. This telemental health study offers promise to address disparities in access to mental health care for people with IDD across diverse racial, ethnic, linguistic, and cultural groups.

**Trial Registration:**

Clinicaltrials.gov (#NCT05336955; Registration Date: 4/20/2022).

## Background

Roughly 40% of those with Intellectual/ Developmental Disabilities (IDD) have mental health needs, which is twice the national average [1, 2]. Unfortunately, equitable access to mental health care for this population does not meet the demand [3]. Untreated mental health symptoms among those with IDD is associated with emergency psychiatric service use and police involvement [4, 5]. For instance, numerous studies have found psychiatric hospitalizations and emergency department admissions are more common for those with IDD when compared to people without IDD [4, 6]. This can result in “boarding,” a situation where the individual spends an extraordinary amount of time (often weeks to months) awaiting an inpatient bed while remaining in the emergency department [7]. Use of emergency room services is not only costly and ineffective, but it is traumatizing to all involved.

The Developmental Disabilities Assistance and Bill of Rights Act of 2000 defines a developmental disability as a condition that is attributable to a mental and/or physical impairment, manifested before the age 22, long-term, results in significant limitations in multiple areas of functioning, and requires specialized supports [8]. Developmental Disabilities reflects a broad range of conditions, such as Autism Spectrum Disorder, Attention Deficit-Hyperactivity Disorder, and Cerebral Palsy [9]. The term developmental disability is often used interchangeably with other labels like neurodevelopmental conditions. However, Intellectual Developmental Disorder is a more specific condition that falls under the umbrella of developmental disabilities. Defined by the 5th edition of the American Psychiatric Association’s Diagnostic and Statistical Manual, Intellectual Developmental Disorder involves impairments in general mental ability that impacts adaptive functioning in social, academic, and self-management skills [10]. This term, created by the American Psychiatric Association, was created to replace the label Intellectual Disability. Given the population described above includes is a highly heterogeneous group, we use the term intellectual/developmental disabilities (IDD) hereafter to reflect all of these persons.

Use of telehealth services has gained prominence as an opportunity to address gaps in mental health services, especially since the COVID-19 pandemic. There is a growing body of research pointing to the effectiveness of telemental health to treat diverse populations for a range of conditions such as depression, anxiety, and eating disorders [11]. However, evidence for the effectiveness of telemental health among persons with IDD is sparse. Telemental health research conducted among this group has predominately focused on the delivery of ABA-based services [12–14], although none of those designs were experimental. A recent virtual mindfulness training program for people with IDD demonstrated positive outcomes, but once again this study was observational [15]. Studies that have deployed randomized designs among persons with IDD have examined cognitive behavioral therapy [16], functional communication [17], and mindset-based interventions [18]. While all these studies demonstrated clinical improvements, their samples were quite small and limited in terms of racial, ethnic, and/or cultural diversity. As a result, a recent systematic review concluded that the current body of research was not sufficient to demonstrate “effectiveness of telehealth-based digital intervention in improving the situation among people with disabilities” [19]. The present study will help fill this gap by evaluating a large scale, systems-based telemental health intervention for a diverse population of people with IDD using a randomized design.

The study will take place in the START (Systemic, Therapeutic, Assessment, Resources, and Treatment) network. START is an evidence-based, cross-systems crisis prevention and intervention model for people with IDD who have mental health needs. First developed in 1988, START has been cited by the National Academy of Sciences and Engineering as a promising model to help overcome disparities in access to mental health care for persons with IDD [20]. In 2008, the Center for START Services was established at the University of New Hampshire Institute on Disability/University Center for Excellence on Disability, College of Health and Human Services.

Several studies have demonstrated positive outcomes associated with the START program. Two studies have shown 1-year pre-post improvements in: (a) service recipient mental health symptoms, as measured by the Aberrant Behavior Checklist; (b) family caregiver perceived quality of care; and (c) decreases in emergency room visits and psychiatric hospitalizations [21, 22]. Notably, these findings were consistent across regions (e.g., Northeast, South, Midwest), populations (e.g., Hispanic and Non-Hispanic), and geographic locale (urban, suburban, rural). A third, much larger study (N = 1,188) examined the timing and outcomes of mental health crises across the national START model [5]. While an increase in contacts was observed in the initial three months of enrollment, which is not surprising given service users and families are enrolled in times of need, there was a steep drop off thereafter with few crises occurring after one year. Importantly, about three out of four crises resulted in maintaining the person in their home.

Like most healthcare services, START had to rapidly transition to telehealth service delivery in response to the COVID-19 pandemic. Preliminary internal data from START indicated that telehealth was an effective method of START service delivery during that time (e.g., 85% of START service users who experienced a crisis maintained their setting). However, the lockdown and other unique societal conditions during pandemic undermine the ability to render these findings generalizable. This study aims to examine the benefits of telehealth as an effective option of service delivery for people enrolled in START and what, if any, methods or conditions contribute to outcomes.

## Methods

### Study aims

The primary aim of this study is to compare the effectiveness of in-person START practices versus START telemental health using a randomized control, non-inferiority design. It is hypothesized that telemental health START will not be inferior to in-person START in the reduction of emergency psychiatric service use, time-to-discharge, improvement in mental health stability and perceived quality of care. This finding will support the use of telemental health practices as a valuable alternative to in-person care.

A secondary aim of this study is to evaluate heterogeneity of treatment response by assessing differences in outcomes across diverse groups, including race, ethnicity, language spoken, rural settings, and level of intellectual disability. It is hypothesized that telemental health will not be inferior to the in-person condition across diverse groups, except for those living in rural settings. We hypothesize improved perceived quality of care will be found among those in the telemental health condition who live in a rural setting, compared to those living in a rural setting who are in the in-person condition, given the known difficulty in accessing in-person services for those living far from care. This finding will support equitable effects of telemental health START practices among diverse racial/ethnic and developmental groups, with potential added value for those living in rural settings.

### Stakeholder engagement

This project brings together stakeholders with a shared commitment to improve telemental health services for individuals with intellectual and developmental disabilities and mental health needs (IDD-MH) from diverse backgrounds and perspectives. We define engagement as the process of working collaboratively with a group of people with diverse characteristics who are linked by social ties, share common perspectives, and engage in joint action toward a particular cause [23]. From design to dissemination of outcomes, our project is grounded in multi-level engagement of a variety of stakeholders in three different ways [23]. First, our Leadership Team includes an individual with IDD-MH, as well as researchers who are family members of people with disabilities. Second, Engagement Teams partnered in the design of research processes and materials to ensure the approach is inclusive of diverse individuals with a range of disabilities, educational, and literacy levels. The Engagement Team meets every other week, and includes people with IDD-MH, family members, START clinicians, and members of our research team. Third, an Advisory Council meets quarterly to provide expert feedback to optimize the importance, feasibility, and accessibility of research processes and outcomes for diverse stakeholders. The Advisory Council includes people with IDD-MH, family members, mental health professionals, researchers with expertise in the mental health and wellbeing of people with IDD, and healthcare policy makers. Finally, throughout the project, we connect with people and families served by START and START providers to build their capacity to engage in research, enhance feasibility, and share what we learn.

### Setting and inclusion criteria

The study will take place in nine states across the US. Within each state, the number of START programs range from 1 to 8 (see Table 1 for details). A total of 500 youth and young adults receiving START services will be enrolled for up to one year or until discharge. Participant inclusion criteria are ages 12–45 years, living in a family setting, and newly enrolled (within 90 days) START service user. The first participant will be enrolled in Spring 2023. The current status of this trial is pending. The study is expected to be completed by 2026.

**Table 1:** Recruitment States and Sites

State	Number of Sites
California	8
Colorado	1
New Mexico	1
New Hampshire	3
New York	5
North Carolina	3
Pennsylvania	1
Tennessee	5
Texas	1

### Intervention

**Fig. 1:**
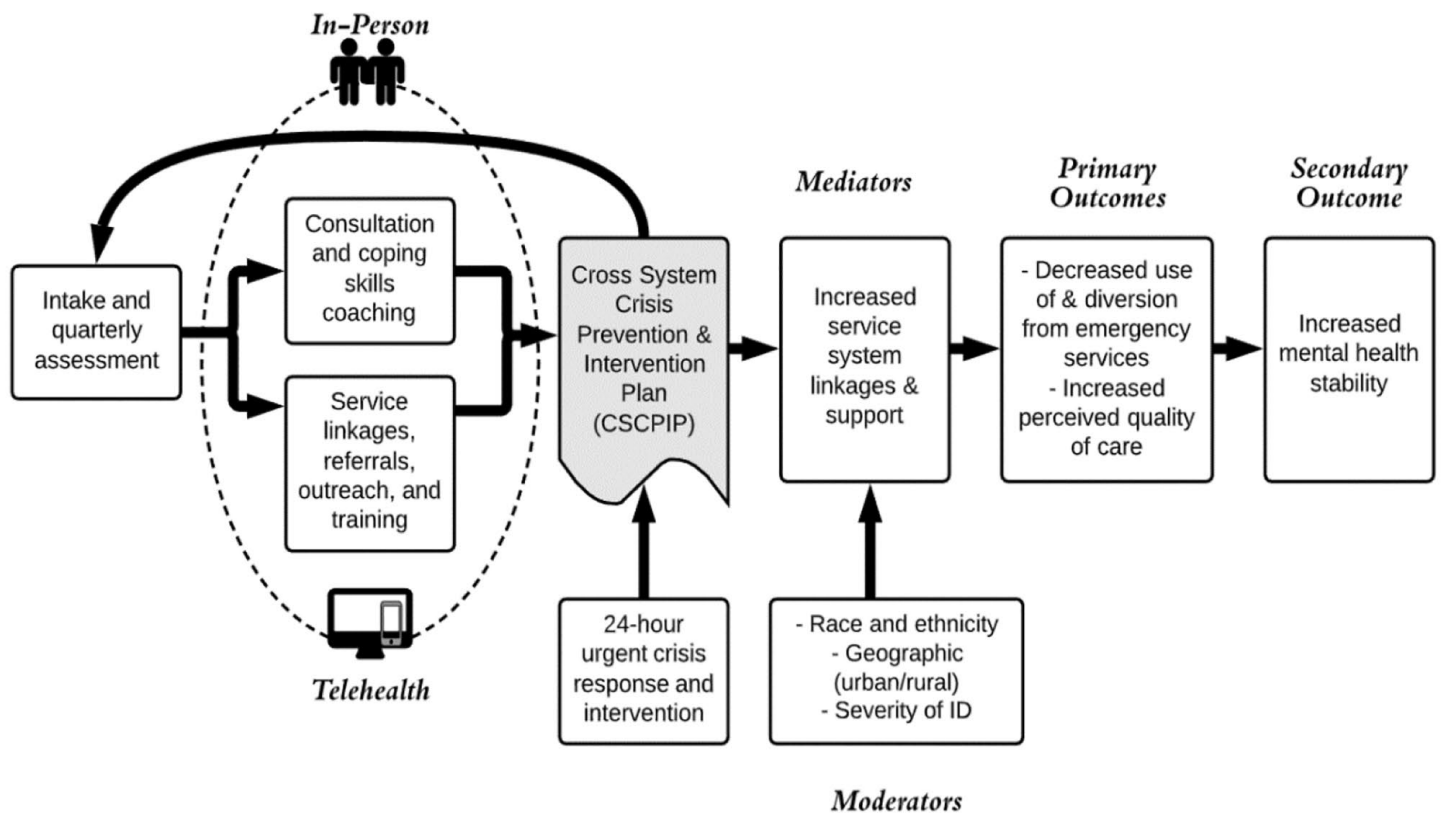
Conceptual Model: START In-person and Telemental Health

START is a complex, multi-component intervention that includes four core elements: (1) outreach, service linkages, referrals, and training; (2) consultation and coping skill coaching; (3) intake and quarterly assessment; and, (4) 24-hour crisis response and intervention [21, 22]. As seen in Fig. 1, these components are based on START’s conceptual model, which is theory-driven and evidence-based. START teams consist of START Coordinators (master’s in social work or counseling) with caseloads of 20–30 people. The team also has a Clinical Director (PhD Clinical Psychologist), a Medical Director (Psychiatrist and/or Child Psychiatrist), and therapeutic coaches (BS/BA).

#### Component 1: Outreach, service linkages, referrals, & training

START teams work with stakeholders to develop and maintain linkage agreements [21, 22]. The purpose of these agreements is to enhance the capacity of the system as a whole and develop partnerships to reduce disparities and gaps in the service array [21, 22]. START coordinators conduct crisis prevention-focused outreach visits with the person and/or their system of care. Examples include training during home- and school-based visits, family caregiver coaching to implement new plans or strategies, and checking in with the person to monitor their level of stability.

#### Component 2: Consultation & coping skills coaching

Mental health service consultation is provided by START clinical and medical directors to prevent and de-escalate crises [21, 22]. Coping skills coaching helps to determine with the person, their family, and the system of care, how to promote well-being and stability. Successful coping and de-escalation practices for the person are incorporated into the Cross-Systems Crisis Prevention and Intervention Plan. All methods have shown value when provided in person. During COVID 19, these services were provided via telehealth with promising outcomes. As part of the current study, separate focus groups with family members, START staff, and START service users were held to better define how to apply these practices via telehealth, and START teams will be trained in order to maximize telehealth effectiveness prior to the randomized control trial.

#### Component 3: intake and quarterly assessment

The START Plan is a custom assessment designed to evaluate the mental health needs of persons with IDD and measure the capacity of the formal and natural support systems [21, 22]. Family caregivers, or the person primarily responsible for day-to-day care of the person, participate in a standardized, 30-60-minute interview conducted by START coordinators. The START coordinator completes the initial START Plan during intake and quarterly thereafter. Quarterly assessment is conducted to inform development or modification of the Cross-Systems Crisis Prevention and Intervention Plan.

#### Component 4: 24-hour urgent crisis response and intervention

START teams have 24-hour, in-person mobile crisis intervention services [21, 22]. Emergency calls come from a variety of sources, including START service users, emergency rooms, service providers, families, and law enforcement. START provides immediate telephonic response and in-person evaluation within two hours of the initial contact.

### Cross-systems crisis prevention and intervention plan

As seen in Fig. 1, the Cross-Systems Crisis Prevention and Intervention Plan provides a comprehensive, person centered, culturally and linguistically accessible roadmap to both prevent crises and intervene in times of acuity. Based on a tertiary care approach to treatment, the plan is designed to deliver a strategy to build capacity to encourage coping skills and address challenges, attain assistance from outside the home/school setting, and respond in times of more urgent need. The plan is developed based on assessments, coaching, outreach, and training provided by the START team in close collaboration with the START service user and the system of support. A member of the START team facilitates the development, review, and revision of the Cross Systems Crisis Prevention and Intervention Plan, with stakeholders engaged throughout the process.

### Comparators

This study compares in-person vs. telemental health START services. For in-person, this comparator will deliver all services and supports to persons with IDD and their families in-person, often in their homes. This is standard START practice. Due to the increased use of telecommunications in the field of healthcare, particularly since the COVID-19 pandemic, individuals in the in-person condition may have large healthcare planning meetings using telecommunication platforms such as Zoom^(TM)^. Use of telecommunications for healthcare planning, such as coordinating care and creating service linkages, will be tracked and assessed throughout the study. The telemental health condition will deliver components 1 & 2 via telephonic or other communication technology and components 3 & 4 in-person. Intake and quarterly assessment (component 3) and 24-hour urgent crisis response and intervention (component 4) will be provided in-person because this is needed to establish therapeutic rapport and ensure the safety of people with IDD and their families.

### Fidelity

All START teams are trained and certified by the Center for START Services for model fidelity. Fidelity to the model is assessed in three primary ways. The first is through extensive training, education, and dialogue. Given the lack of graduate education that addresses the mental health aspects of IDD, ongoing professional development, training, and quality review are needed to ensure competency across START teams. The Center for START Services also provides clinical apprenticeship opportunities, practice improvement groups, community training, and cross-systems engagement. The second approach is to monitor fidelity to START practices through the Center for START Service’s database. Third, is ongoing research and evaluation to continuously assess and improve START methods via analysis of outcomes as reported in the START database, also termed SIRS (see below).

#### Data capture and entry

START employs total quality improvement approaches including the START Information Reporting System (SIRS), a robust and secure data collection, reporting, and evaluation infrastructure. START teams are trained to accurately enter data into SIRS using a comprehensive data dictionary. The quality and frequency of data entry is closely monitored by the Center for START Services, where data are housed. Data from SIRS will be used for this analysis.

### Assessments

Emergency service use and time-to-discharge will be assessed continuously throughout the 1-year study time line. All other assessments will be done at baseline, 6 (midpoint) and/or 12 (outcome) months, except for the START plan (which is conducted quarterly), the Family Experiences Interview, and the Person Experiences Interview (conducted at pre-post).

#### Mental health crises and emergency service use

The primary outcome for this study is use of and diversion from emergency mental health services. This includes emergency department visits and in-patient hospitalizations, including length of stay and police/jail involvement, and number of 24-hour START in-person crisis contacts. All of these data are gathered by the START Coordinators in the SIRS database.

### Perceived quality of mental healthcare

#### The family experiences interview schedule

The second outcome is family caregiver evaluation of their experiences with the mental healthcare system. This is assessed via the Family Experiences Interview Schedule (FEIS), a widely used semi-structured interview [24]. Baseline FEIS interviews are conducted by the START coordinator in the ordinary START intake process. Follow-up interviews will be conducted by an individual who was not involved with the person’s care. Interviews take approximately 20 min to complete. Three FEIS subscales will be used. The first (9 items) assesses family members’ appraisal of their own involvement as partners in treatment, the second Sect. (7 items) assesses perceived quality of services provided to the recipient, and the final Sect. (4 items) elicits feedback about how well the mental health system responds to the needs of family caregivers. Previous research across multiple samples has shown the FEIS and these subscales to be reliable (Cronbach’s α = 0.92) and internally valid [21].

### The person experiences interview schedule

To ensure our study includes outcomes reported from the perspectives of people with IDD, we are designing a novel patient-reported outcome measure called the Person Experienced Interview Schedule (PEIS). This measure is critical since the FEIS relies on family caregiver informants who may not share the same perspective as youth and young adults with IDD. The PEIS will be adapted from the FEIS. We have chosen to adapt the FEIS because: (1) it is brief; (2) it focuses on perceived quality of mental healthcare, a critical outcome of this study; (3) to our knowledge, no measures are available for this outcome that can be used for persons with IDD; and (4) it directly aligns with the goals of the START model. Our adaptation process is following best practice, patient-reported guidelines put forth by COSMIN. Details of this measure will be reported in forthcoming publications. Its design and ethical use has been approved by the governing Institutional Review Board.

#### Mental health symptoms

The Aberrant Behavior Checklist – Community Version will be used to measure problem behaviors associated with mental health symptoms [25, 26]. The ABC is a well-known and psychometrically robust measure that was designed specifically for persons with ID. The Irritability, Lethargy, and Hyperactivity subscales will be the focus of this study. Family caregivers serve as the informant.

Mental health stability, as measured in the START Plan, is comprised of (1) intensity of mental health symptoms and (2) risk to self or others. Symptoms encompass 16 items, such as verbal and physical aggression, suicidality, anxiety, depression, psychosis, and trauma-related symptoms, all of which are rated on four-point scale from “no concern” to “high concern.” Risk to self or others is determined with 7 items, which reflect behavioral acuity, as rated on a four-point scale of “rarely” to “often.” Raw scores are summed to create the composite score, associated with four categories of instability: stable, low, moderate, and high.

#### Time-to-discharge

The final outcome is time to discharge. Discharge occurs when the person no longer is at high risk for mental health emergency service use. Discharge is determined in collaboration with the person, their family caregiver, and their interdisciplinary team. It is an indicator of mental health stability. This indicator will be measured as the difference in days between START admission and discharge.

### Masking, randomization, and data safety monitoring

Based on feedback from family and provider stakeholders gathered during the development of this proposal, participants will not be masked. Randomization will occur at the individual level using a random number generator in blocks of two to ensure that participants within the settings are allocated equally to conditions. Randomization will be conducted by an independent party and study coordinators will be unaware of the randomization allocation until participant enrollment. Allocation will be revealed via a blinded excel file after participant enrollment. This study adheres to the SPIRIT guidelines.

We have developed an ethically approved data safety and monitoring plan. This plan involves obtaining informed and written consent from the guardian(s) as well as assent from all participants, by the designated research coordinator, and safely storing data and restricting unauthorized access to identifiable participant information. Additionally, this plan outlines the protocol for responding to any adverse events/reactions to the randomly assigned treatment options. While this study is low-risk, there is a limited possibility that participants in this study will experience unknown effects. Therefore, the study team will closely monitor the acceptability of the procedures and seek to understand any concerns from participants and guardian(s) to ensure they are addressed in a timely matter. If a formal concern is voiced by a participant or by their guardian(s), which is possible at any time (via provided study contact information), it will be immediately addressed to the best of the study team’s ability and will also be reported to the ethics board. Discontinuation of the study protocol will be made at the participants’, ethics board, and/or monitoring boards’ request. No specific modifications are considered at the present time.

A Data Safety and Monitoring Board (DSMB) will monitor all clinical outcomes and adverse events. This includes hospitalization(s), changes in mental health presentation, and contact with the START crisis response team. Since START is a crisis prevention and intervention program, it is designed to handle clinical events through prespecified protocols and a robust clinical team. The DSMB will be led by an independent team made of three psychologists, and a family advocate. No members of the DSMB team have direct involvement with the study or any conflict of interest (COI) with the investigators conducting the study. No interim analyses or stopping rules have been defined by the DSMB, as of yet.

### Non-inferiority design

This study will employ a non-inferiority design. Non-inferiority trials permit a ‘new’ treatment to be considered efficacious if treatment effects fall within a pre-specified margin (the non-inferiority [NI] margin) of the effects identified in the reference treatment [27]. In this study, the ‘new treatment’ will be the telemental health condition, while the in-person condition will serve as the ‘established/reference treatment’. The merits of telemental health that justify consideration as an alternative to the standard of care include increased accessibility and lower cost. The merits of telemental health are assumed to be valuable enough that, even if effects fall slightly short of the reference treatment by a clinically tolerable amount, it is a viable alternative to the standard of care when circumstances would indicate its use [28].

The NI margin has been set for the FEIS, given its primacy in outcome. A 7-point change in the total FEIS score, which is equivalent to a standardized delta of 0.5, will identify the lower threshold for clinically meaningful difference [21]. Thus, a 99% confidence interval for the slope for the group assignment variable will be constructed and its lower limit will be compared to the − 7.0 non-inferiority margin. The 99% confidence interval, reflecting the α cutoff of 0.008, was selected to account for multiple comparisons, which will be determined post-hoc via the Benjamini Hotchberg procedure [29]. The non-inferiority margin for emergency service use will be a 38% reduction in the probability of a START crisis contact between START in-person and START telemental health. Last, we have identified a five-point change in the ABC irritability subscale as the non-inferiority margin. This study was registered on April 20th, 2022 on clinicaltrials.gov (NCT05336955; https://clinicaltrials.gov/ct2/show/NCT05336955 ).

### Statistical analyses

The efficacy analyses will be conducted as both intention-to- treat (ITT; “as allocated”) and per protocol (PP; “as-treated”) since ITT can bias towards the null, leading to false claims of non-inferiority, and PP can fail to preserve the balance inherent to randomization (30, 31). For the analysis, the first step will be to examine demographic and clinical characteristics across study conditions to assess that the randomization worked as expected. If significant sociodemographic differences emerge, these variables will be included in the multivariate analyses since baseline adjustment has been shown to increase precision in randomized control trials (32). All analyses, for the factorial design, will be examined using a series of multilevel (or “mixed” effects) general linear regression models that account for clustering (via a random intercept) across sites. For these models, the independent variable of interest will be telemental health versus in-person START. The dependent variables will be calculated as change scores/differences since baseline. Two-way (Intervention group x time) interaction terms will test the primary study aim. Three-way (Intervention group x demographic population x time) interaction terms will test the secondary study aim. Effect sizes will be calculated using Cohen’s *d*. To accommodate increased false discovery rate, the study will adjust the significance threshold using the Benjamini-Hochberg procedure. This procedure is conducted after all tests are concluded; α = .01 will be assumed for the power analysis.

### Power analyses

Using data from Kalb et al. 2019 [21], we calculated the sample sizes needed to have 80% statistical power to detect the above-mentioned non-inferiority margins with α set at 0.008. For changes in total FEIS scores from baseline to follow-up between the study arms, this study requires N = 128 per group. For a 38% reduction in the probability of a START crisis contact between conditions, this study requires N = 250 per condition. For changes in the mean Aberrant Behavior Checklist irritability scores, this study requires N = 100 per condition.

## Discussion

To our knowledge, this will be the first trial of an established program to provide telemental health crisis services for the IDD population. The study will be executed by an interdisciplinary team of experts that includes stakeholder partners with lived IDD-MH experience. Understanding the benefits of specific telemental health methods has important implications to the design of interventions, within and outside of START. This telemental health study also offers promise to address disparities in access to mental health care for people with IDD across diverse racial, ethnic, cultural, and linguistic groups.

### Responding to cultural context

It is important to recognize that all research is conducted in a cultural context. This includes the question or focus of the research, the research participants and the communities in which they live, the research institution/organization, and the research team. Addressing the cultural context requires a fundamental change in how research is designed, conducted, translated, and disseminated in partnership with diverse stakeholders in general, and for this study, persons with IDD-MH experience in particular.

Persons and their families who are enrolled in START are members of diverse racial, ethnic, cultural, and linguistic groups who reside in varied geographic locales throughout the US. Our study is employing principles and practices of cultural and linguistic competence in order to be responsive of their diverse needs and identities [33, 34]. For instance, the study team: (1) pre-specified facilitators and barriers that have cultural implications (e.g., race, ethnicity, languages spoken, and other cultural factors); (2) acknowledged the diversity across populations by disaggregating data according to race, ethnicity, language spoken, level of intellectual disability, and other cultural factors; (3) employed inclusive practices in study design and reporting; and (4) will analyze heterogeneity in treatment responses and outcomes across prespecified racial, ethnic, linguistic, and cultural groups.

### Advisory council and engagement team

Partnerships with stakeholders at all phases of the research process enhances our study in many ways. First, designing study materials in collaboration with stakeholders, such as plain language informed consent videos, FAQs about the study, and recruitment flyers helps to ensure people with IDD and family members can make an informed decision about their participation. Designing accessible materials can empower people with IDD as well as guardians with diverse educational levels, literacy, or familiarity with research to make an informed choice about participating in this study. Second, clear understanding about the research study during the enrollment process can enhance retention for the duration of the study. Retention is a crucial aspect of this study design, and traditionally can be challenging in long-term studies, or studies with populations at high risk of crisis such as START users. Third, partnership from the ground up with the community means the proposed interventions and outcomes examined are more likely to be relevant for ‘real-life’ service users, including people with IDD enrolled in START and their families, as well as START providers. Insights shared along the way ensure we are measuring outcomes that matter most to people, and the interventions and tools used to reach those outcomes are more likely to be acceptable and adopted in future practice.

In summary, this study seeks to understand the efficacy of telemental health delivery for crisis intervention and prevention services for persons with IDD. While some services and treatments may not require extensive attention to the method of delivery, for people with disabilities this is likely not the case. If our results support including non-inferiority of telemental health to in-person START, these findings will increase the confidence in telemental health service delivery to persons with IDD. It will also increase the prospect of making telehealth more accessible to a wider population of service users. The impact of such would be far-reaching, and enable families who encounter barriers to receiving in-person services to access evidence-based intervention.

## Data Availability

Voluntary, informed, written consent will be obtained during recruitment for the publication of the dataset. The appropriately de-identified study materials and data will be made available publicly available upon completion of the trial. This includes individual-level study data from the final trial dataset, data dictionaries, statistical code, CONSORT (Consolidated Standards of Reporting Trials) diagram (e.g., flow charts), data forms (where appropriate), analytic plan, and study protocols. These data will be available in perpetuity by 2026. These data will be made freely available here: https://www.icpsr.umich.edu/web/pages/pcodr/.

## Abbreviations

IDD: Intellectual/developmental disabilities
START: Systemic, Therapeutic, Assessment, Resources, and Treatment
AC: Advisory Council
CSCPIP: Cross-Systems Crisis Prevention and Intervention Plan
SIRS: START Information Reporting System
FEIS: Family Experiences Interview Schedule
PEIS: Person Experienced Interview Schedule
NI: Non-inferiority
ITT: intention-to- treat
PP: per protocol
CONSORT: Consolidated Standards of Reporting Trials
DSMB: Data Safety and Monitoring Board
PCORI: Patient Centered Outcome Research Institute
